# Alcohol in Relation to Therapeutics

**Published:** 1906-06-02

**Authors:** 


### The Hospital

A Journal of

The Medical Sciences and Hospital Administration.

Vol. XL.—No. 1,027. SATURDAY, JUNE 2, 1906.

### Alcohol in Relation to Therapeutics.

The alcohol question is one which has many aspects and many relationships, and probably on most of these there exist sharp differences of opinion even between men essentially moderate and fair-minded. The medical profession is inevitably closely concerned in these various controversies, both because its members are assumed to be able to speak with authority on the physiological action of alcohol, and because upon them, in numerous individual instances, falls the decision of advising or forbidding its use. No one will pretend that universal agreement marks the opinion of the profession on the questions which arise in the discussion of these points, and there can be no doubt that men of equal honesty and equal capacity hold diametrically opposite views. Like many other problems of physiological chemistry, the action of alcohol upon the tissues and organs of the human body is not easily placed on a confident foundation, and even when observations of fact are generally accepted, rival and conflicting interpretations sometimes offer themselves. What is true in these respects in relation to health is even yet more true in regard to disease. Here the conditions are still more complex and the anxieties of decision still more keen. It must be remembered also that experimental observations conducted for scientific purposes are never conditioned by exactly the same circumstances as those which exist in the case of disease, and consequently the last word regarding the therapeutic value of alcohol cannot rest with the physiologist. The facts contributed by the practical physician are an essential part of the discussion, and their weight and force must be treated with not less respect than that paid to the more precise, and in some sense more attractive, figures of the laboratory. There is still another factor to be recognised. It is provided by the fact that in the alcoholic preparations usually employed in the treatment of disease, and more particularly of acute disease, alcohol is by no means the sole agent. Volatile ethers and other allied substances have here to be reckoned with, and thus conclusions relating to the effects of pure alcohol do not afford an exact parallel. All these considerations, it will be acknowledged, bear on the points at issue and show that the decision is not to be reached by an easy and confident formula. In stating them, however, our object is not to endeavour to make converts either on one side or the other, but to plead that a question so involved shall be kept within the limits of scientific discussion and free from the invasion of popular prejudice and passion. Our reference here is, of course, rather to the therapeutic uses of alcohol than to its social or economic influences. Concerning these latter the medical man has much the same rights and relationships as has the ordinary citizen, but as regards the use of alcohol in disease he sustains an almost undivided responsibility. Doubtless most members of the profession have practically settled the question one way or the other as a result of their individual experiences, and it is not too much to say that the preponderance is enormously in favour of the view that alcohol and its allies are in various circumstances valuable, or even essential, members of the materia medica. For the moment, we are not contending that such a conclusion is sound or the reverse; but we do urge that it is a fact of great significance, that it is a necessary element in the discussion, and that if it is to be upset this must be accomplished by fair argument and demonstration and not by the pressure of popular and ignorant prejudice. The time has long gone by for the settlement of scientific and medical questions by an appeal to orthodoxy or by the voice of some *ex cathedra* authority. But to replace autocracy by mob rule would be but a sorry revolution. The theory of the profession excludes both the one and the other and allows honest men freedom to frame their own judgments without fear either of frowns from above or tumult from below. Applying this principle to the alcohol question we venture to urge both the majority and the minority strictly to confine discussions on the problems of therapeutics to the professional arena. Every medical practitioner is free to use or to abstain from using alcohol in the treatment of his patients, and he is equally free to press his opinions and the reasons for them on his professional brethren. To go outside these opportunities and to protest before the public has this peril, that it drags into the discussion those who cannot possibly appreciate the points at issue. Hence we see such ill-advised suggestions as a recent proposal of a lay member of a hospital board that a medical officer should be replaced by "another" who would order neither wines nor spirits, this persecuting proposal being defended by a reference to a hospital which proclaims its "temperance" principles. For ourselves we believe the general view of the profession to be based on well-established facts, but we desire neither to persecute those who differ from us nor to suggest their inferiority. To the facts and arguments they submit we are ready to give attentive heed, but these will not be strengthened by an alliance with popular clamour.

